# Cognitive Training for Very High Risk Incarcerated Adolescent Males

**DOI:** 10.3389/fpsyt.2020.00225

**Published:** 2020-04-15

**Authors:** Abby Rowlands, Melissa Fisher, Jyoti Mishra, Mor Nahum, Benjamin Brandrett, Michael Reinke, Michael Caldwell, Kent A. Kiehl, Sophia Vinogradov

**Affiliations:** ^1^School of Nursing, University of Maryland Medical Center, Baltimore, MD, United States; ^2^Department of Psychiatry and Behavioral Sciences, University of Minnesota, Minneapolis, MN, United States; ^3^Department of Psychiatry, University of California, San Diego, San Diego, CA, United States; ^4^Posit Science Inc., San Francisco, CA, United States; ^5^Department of Psychiatry, University of California, San Francisco, San Francisco, CA, United States; ^6^Medical School, University of Minnesota, Minneapolis, MN, United States; ^7^Department of Psychology, University of Wisconsin, Madison, WI, United States; ^8^Department of Psychology, University of New Mexico and Mind Research Network, Albuquerque, NM, United States

**Keywords:** cognitive remediation, adolescence, conduct disorder, violence, cognition

## Abstract

**Objective:**

Persistent violent and antisocial behavior, as manifested in conduct disorder (CD) traits, are associated with a range of cognitive deficits. Individuals with more severe cognitive deficits are more likely to commit violent crimes. Currently, no treatments target improving cognition in high-risk CD youth. This pilot study tests the feasibility and efficacy of delivering intensive tablet-based cognitive training (CT) to adolescent males incarcerated in a youth maximum-security prison.

**Methods:**

Participants were fourteen adolescent males, diagnosed with CD. All participants completed up to 30 h of unsupervised, intensive, adaptive CT exercises that targeted multiple neurocognitive domains, as well as a battery of standardized neurocognitive measures and computerized assessments at baseline and post-training.

**Conclusions and Implications for Practice:**

At baseline, participants exhibited significant impairments on neurocognitive measures, relative to age-matched healthy controls. Twelve participants completed training and showed evidence of target engagement, as indexed by improvement in cognitive processing speed. Significant gains were observed in measures of global cognition, with additional gains in cognitive flexibility at trend level significance. Improvements in these measures were positively related to total training time. In summary, both assessments and intervention appear to be feasible, tolerable, and acceptable in incarcerated youth. Intensive CT shows preliminary efficacy in improving neurocognitive performance in key domains, with large effect sizes, and significant performance improvement associations with the time in training.

## Introduction

Violent criminal behavior carries high personal and societal costs, but only a small proportion of offenders commit the majority of all violent crimes (1, 2). These offenders typically display antisocial behavior from a young age—showing “callous-remorseless” traits as well as “impulsive-antisocial” traits that emerge in childhood and adolescence and continue through adulthood (3, 4). High levels of these callous-unemotional (CU) and conduct-disorder (CD) traits in adolescence are associated with life-course persistent antisocial behavior (5–7). It is now also well-established that persistent violent and antisocial behavior is associated with a range of neurocognitive abnormalities (8–22).

Broadly speaking, the neurocognitive abnormalities seen in persistent violent and antisocial behavior occur in prefrontally-mediated higher-order cognitive operations and in social-affective processing. For example, deficits are seen in sustained attention, working memory, and cognitive control (i.e., impairments in allocation of attentional resources, task-switching, error-monitoring, response inhibition, decision-making, and adaptation to stress), as well as in prefrontal modulatory control over reward processing and emotion processing (23–32). Individuals are noted to spend less time thinking before attempting to solve a problem, which may be indicative of underlying impulsivity (33). Deficits are also seen in processing basic social and emotional cues (such as fear and pain), along with impaired empathy and theory of mind abilities (14, 28, 30, 33–36). Furthermore, it has been proposed that deficits in social information processing are influenced by processes of executive function (37).

Individuals with more severe cognitive deficits show higher CU/CD traits and are more likely to commit violent crimes (15, 16). Cognitive impairment also shows evidence of being associated with recidivism. Impaired executive function appears to predispose to recidivism among young first-time male offenders with CD, and lower scores on the Iowa Gambling Task (indicating lowered prefrontal inhibitory control) predict higher recidivism rates at 6-month follow-up (17, 38). Low, relative to high, error-related activity in the anterior cingulate during a Go-NoGo task predicts nearly four times the re-arrest rate within four years in adults (9). In adults, differences in performance (associated with psychopathy or antisocial personality disorder) on decision-making tasks are predictive of higher rates of incarcerations and arrests (39, 40).

Taken together, the emerging evidence suggests that developing a treatment to improve cognition—perhaps particularly improvements in inhibitory control and error-monitoring—may in a downstream manner, ameliorate some maladaptive behaviors in high-risk CD youth. Even only modestly improved cognitive capacities, might enable some individuals to make better use of educational and vocational rehabilitation programs, thereby supporting better decision-making and more adaptive functioning in the community. It is possible that altering behavior in such a manner could provide further benefit by fostering the development of positive life expectations.

Given the many challenges that researchers face working with ultra-high risk populations, particularly within Department of Justice institutions, it is understandable that a gap in the literature exists. Some focus has been put on the feasibility and efficacy of group psychiatric intervention programs, Cognitive Behavioral Therapy and Mindfulness Training, however these studies do not include children or adolescents and are not specific to a particular psychiatric diagnosis (41–43). A significant percentage of research done in correctional settings is dedicated to addressing addiction and substance abuse, as well as physiological disorders such as diabetes, Human Immunodeficiency Virus and Hepatitis (44–48). While these studies are extremely valuable in their own right, they fail to directly address another primary cause of recidivism such as violent and antisocial behavior. Correctional settings are increasingly becoming primary settings for psychiatric rehabilitation research, therefore this pilot study offers important preliminary results in support of increased effort and attention from contemporary researchers to further investigate vulnerable populations in such environments.

Recent data suggests that first-time adolescent offenders who subsequently partake in less crime tend to develop improved expectations, and those with higher expectations commit fewer crimes (49). Thus, investigation of cognitive treatments for this population are imperative. To this end, we performed a pilot study of computerized, intensive, targeted cognitive training in 14 adolescent males incarcerated for violent crimes in a youth maximum security prison setting (Mendota Juvenile Treatment Center, MJTC). Our primary goals were: 1) To elucidate the baseline profile of cognitive impairments in a sample of MJTC participants, using both standard neuropsychological measures as well as novel online cognitive assessment tasks; and 2) To determine the feasibility, tolerability, and acceptability of tablet-based intensive cognitive training exercises. Given that this was a pilot study with no control intervention group, our remaining goals were exploratory, and we sought to examine 3) target engagement as a result of training (improvement in speed of processing); 4) the preliminary efficacy of cognitive training; 5) the associations between target engagement, neuropsychological gains, and “dose” of training.

## Methods

### Participants

#### Overview of the MJTC Population

This study took place over an eight-week time period at MJTC. Sixty percent of the youth incarcerated at MJTC have been charged with three or more crimes against persons; ~50% are committed for a violent felony offense, and ~50% have hospitalized or killed a victim. Youth mainly come from economically disadvantaged, violent, or disrupted homes. Fifty percent became involved in crime before their 10th birthday. The gender and racial/ethnic composition is 100% male, approximately 51% African American, 38% Caucasian, 9% Hispanic, and 2% Asian or Middle Eastern. The average IQ is 85, and the mean grade achievement level is 5^th^ grade. Approximately 95% of MJTC youth have a primary diagnosis of CD, 75% have ADHD, 50% have a concurrent mood disorder, and 5% have a diagnosis of schizophrenia or bipolar disorder; approximately 70% have had a substance use disorder. Medication for comorbid disorders was on a case-by-case basis as determined necessary by Mendota treatment staff. Medication type or doses were not changed for the purpose of participation in the study.

#### Pilot Study Participants

Participants were 14 male adolescents incarcerated at MJTC, ages 13–17 (Mean=15.50, SD=1.40), with an average reading grade level of 4.21 (SD=2.46), and average IQ of 79.29 (9.29). Eligibility criteria for study inclusion were a DSM-IV chart diagnosis of CD (diagnosed by MJTC mental health staff upon entry to MJTC), incarceration in the MJTC program for at least 2 months, no active psychosis, and behavioral stability prior to joining the study (not determined to be actively aggressive or behaviorally disruptive by MJTC staff). In addition to a diagnosis of CD, comorbid clinical psychiatric diagnoses included: ADHD (N=11), Mood Disorders (N=9), Substance Use Disorders (N=6), Psychotic Disorders (N=1), Attachment Disorders (N=2).

### Procedures

#### Enrollment

Under the on-site supervision of MJTC correctional and mental health treatment staff, referrals were made to the research team. Fourteen MJTC youth were approached over an eight-week time period, met the eligibility criteria, underwent informed consent (as minors, giving assent with patient advocate present), and participated in baseline assessments. Assessments were conducted over two sessions of 45–60 min. Targeted cognitive training *via* tablets (i.e. iPads) was then offered during 1.5-h time blocks on weekday mornings or afternoons during “down time” when participants were alone in their cells without other obligations or activities. During these cognitive training sessions, a research associate was available to answer questions related to the exercises. At the completion of training, or at the end of the eight-week study period (whichever came first), participants completed post-training assessments.

Participants were paid $5 per session for assessments, and $0.50 an hour for training; payments went to their canteen accounts each time they earned $2.50, as per institutional policy. The reward and payment system was approved and compliant with MJTC policies and the MJTC Institutional Review Board.

#### Cognitive Training

For the purposes of this pilot study, we aimed to deliver 10–30 h of training of auditory speed of processing, auditory and visual attention, and auditory and visual working memory and cognitive control exercises *via* tablet [Brain HQ, Posit Science Corporation (PSC)] in the eight-weeks available to us for implementation of the study. Based on our prior cognitive training studies, we have determined that for individuals with diagnosed axis-1 psychiatric disorders, 30 h of training is able to produce significant outcomes improvements (50–52). In other studies of targeted cognitive training, especially with respect to intensive training in a singular cognitive training, ~10 h of training has also demonstrated significant target engagement and cognitive transfer (53, 54). Thus, based on the diversity of cognitive training components, 10–30 h of training has been found to be feasible and efficacious.

The 9 training exercises included in the training suite (see complete list and exercise descriptions in **Supplemental Table 1**) are tablet versions of the original Brain Fitness Program (BFP) and InSight training suites [see Fisher et al. (50) and Chen et al. (55) for BFP and InSight exercise descriptions]. Over the course of training, exercises are gradually introduced, and collectively emphasize improvement in the learner’s speed of processing of basic cognitive abilities through repetitive practice and implicit learning. Exercises require that the learner attend, make fine discriminations, hold in working memory, and perform a response action, to specific components of speech as well as to salient visual-spatial information.

**Figure 1:**
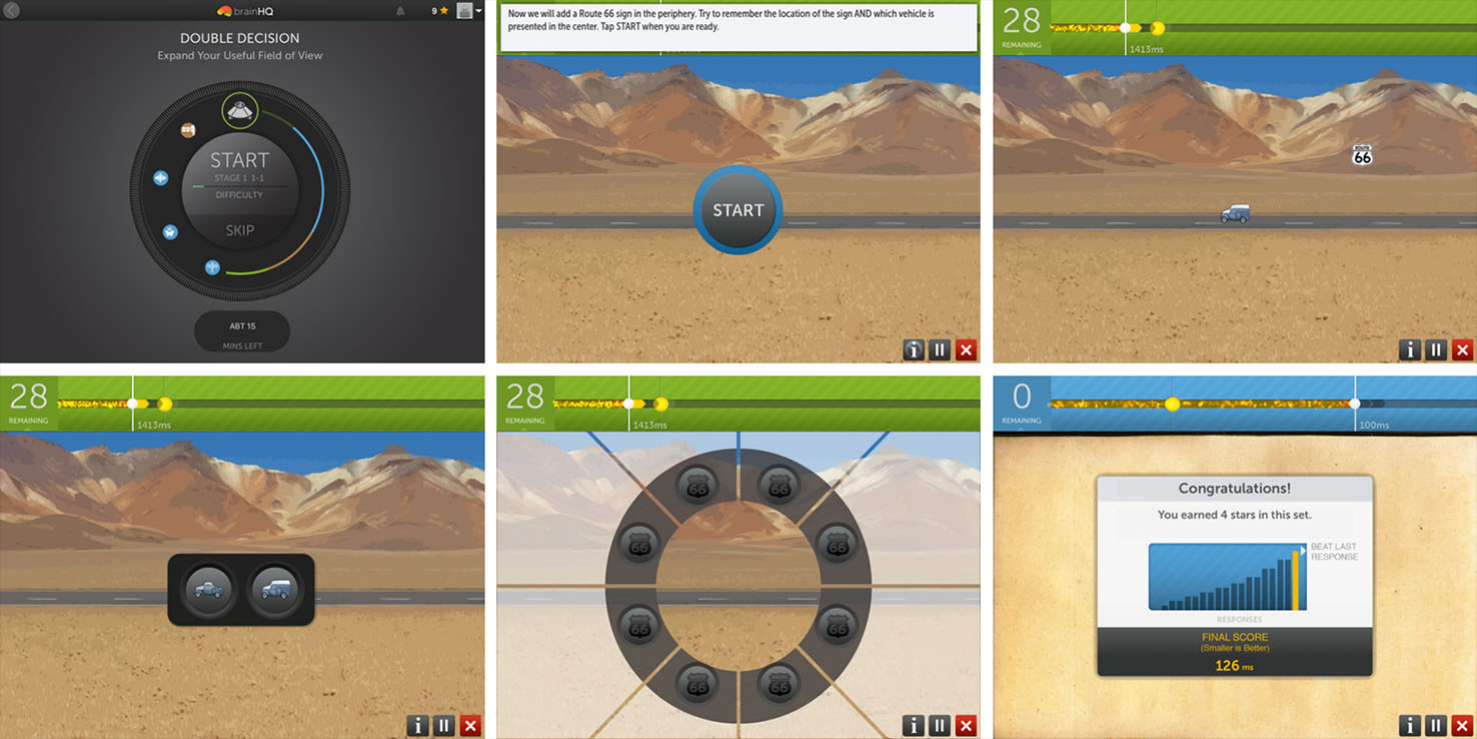
An example of an iPad Brain HQ training exercise, Double Decision. Top row, left to right: Training starts with a “wheel” display of today’s training exercises. Then, exercise instructions are displayed. In a Double Decision trial, the user will need to correctly identify the central object (one of two cars) and correctly select the peripheral location of the sign “route 66”, as they are quickly flashed on the screen. Bottom row, left to right: response screens (central object followed by peripheral object). At the completion of the exercise, a results screen is displayed, showing the user’s score and number of stars earned.

Within a given training session, exercises continuously adjust difficulty level to user performance to maintain an 80% correct performance rate using adaptive algorithms (56). Correct trials are rewarded with points and animations, whereas incorrect trials are indicated by a “thump” noise as error feedback. At the completion of an exercise, the users are prompted with a screen showing them their score (see **Figure 1**). In the course of training, exercise configurations gradually become more challenging and difficult. Participants were asked to complete 60 min of training per session. Each exercise ranged from 2.5 to 4 min in length. At the completion of each training session, training data was securely transferred online to a secure server maintained by PSC.

The tablets used by subjects to complete their training were in a passcode-controlled “Guided Access” mode, which limits the device to a single application, restricts users from exiting the application, and disables areas of the screen that are not relevant to the task in the application. Each user had a unique password-protected login that was used for training purposes only.

#### Assessments

In order to determine tolerability and acceptability of different methods of neurocognitive assessments in this subject sample, we assessed 7 cognitive domains using well-validated standard paper/pencil and computerized neuropsychological tests, and re-assessed 4 of the 7 same domains using a novel on-line computerized cognitive (CC) assessment battery (PSC-CC, **Table 1**). All raw scores were converted to age-adjusted T-scores using published healthy control normative data for the standard neuropsychological measures. Age-matched, healthy control normative data for the PSC-CC battery was collected from adolescents attending school in the San Francisco Bay area. Parents/legal guardians of these participants provided written informed consent, and adolescents provided verbal assent for this data collection approved by the University of California San Francisco Institutional Review Board. Participants in this control sample were screened to not have any neuropsychiatric disorder and not taking any psychotropic medications at the time of the assessment. Speed of processing and cognition composite scores were computed as the average T-score across speed of processing measures and all standard neuropsychological tests, respectively (see **Table 1**). Alternate forms of NAB Mazes were administered and counterbalanced at baseline and post-training.

**Table 1:** Neuropsychological measures and computerized cognitive tests used to assess cognition.

Cognitive Domain	Standard Neuropsychological Measures	Posit Science Inc. Automated Computerized Cognitive Tests (PSC-CC)
**Attention**	CogState One-Back Task (computerized)	A TOVA-like task. Sustained visual attention task (respond to visual target, ignore distractors)
**Speed of Processing**	Trails A, BACS Symbol Coding, D-KEFS Color, and Word Reading	Sound Sweeps. An auditory perceptual processing speed task (time-order judgment task for two FM sound sweeps)
**Working Memory**	UMD Letter Number Span	A visuospatial working memory task (remember spatial location of an array of objects)
**Verbal Learning**	CogState International Shopping List Task (computerized)	N/A
**Cognitive Flexibility/** **Switching**	Trails B	A cognitive control task (“Task Switcher”), in which the user needs to use a rule (color vs. shape) to guide response to the target. The rule changes each trial
**Problem Solving**	NAB Mazes	N/A
**Response Inhibition**	D-KEFS Color Word Interference	The cognitive control task described above contains some elements of response inhibition (subjects must inhibit use of rule from prior trial)

We were provided access to reading and IQ level, which were assessed by MJTC staff upon each participants’ entry to MJTC. Reading level was assessed with the Peabody Individual Achievement Test (the standard test used by the Madison, WI School District) and IQ was estimated with the Matrix Reasoning and Vocabulary scales of the Weschler Abbreviated Intelligence Scale.

### Analyses

We performed data analysis on all subjects completing baseline and post-training assessments regardless of hours of intervention. All variables were screened and normally distributed after winsorizing of outlying values (± 2.5 SD from the mean). Paired Samples T-tests were used to test for improvement in cognition from baseline to post-training. Pearson correlations were used to test the association between hours of training and the change in Auditory Processing Speed and the cognition composite scores.

## Results

### Feasibility, Tolerability, and Acceptability

All 14 subjects began training; two dropped out after completing less than 2 h, one due to behavior issues not related to study participation, and one due to discharge from the facility. The exercises were well tolerated and acceptable by the remaining 12 subjects. Participants were able to tolerate the two assessment sessions of 45–60 min at baseline and post-training including the PSC-CC assessment battery.

Subjects trained 1–5 times per week, for sessions that were an average of 77 min in length (SD=62.56, range of 21–210 min). Nine boys showed high adherence to the training schedule, training for at least 40 min per week. By the end of the eight-week study period, subjects had trained for a total of 8.1 h on average (SD=8.31, range of 1-27 hours). Subjects were involved in training for 10–30 h over eight weeks.

### Baseline Cognitive Performance

**Figure 2:**
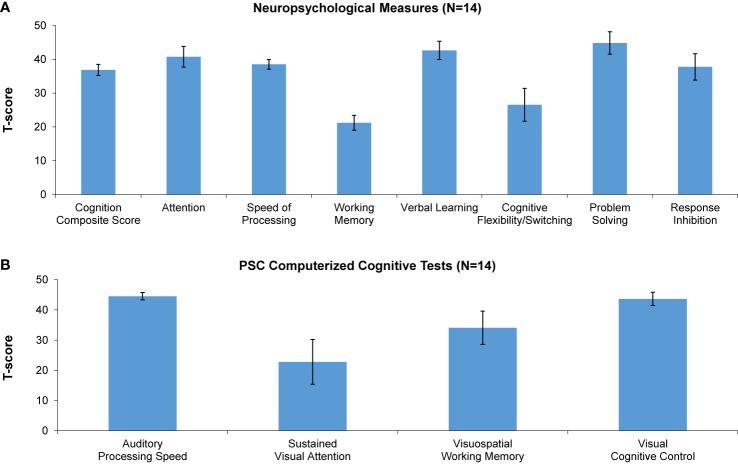
Baseline performance (T-scores) on neuropsychological measures **(A)** and Posit Science automated computerized cognitive tests **(B)**. A T-score of 50 denotes performance in age-matched healthy controls. * denotes significantly lower performance of incarcerated youth relative to healthy controls.

Compared to age-matched healthy controls, MJTC youth were impaired across a range of measures at baseline, generally performing at least 1 SD below, and in some instances 3 SD below, age-matched norms. Baseline data are shown in **Figure 2**. The greatest impairments were found on standard neuropsychological measures of working memory and cognitive flexibility/task switching, and on the PSC-CC measures of visual attention and visuospatial working memory.

### Evidence of Target Engagement and of Preliminary Efficacy

**Figure 3:**
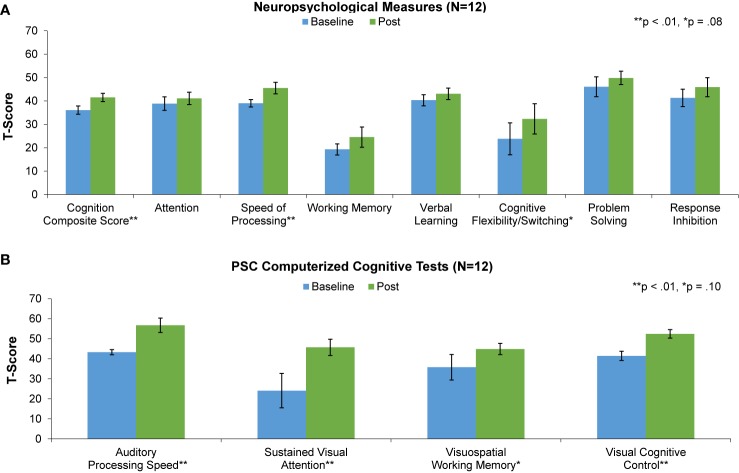
Baseline (blue) and post-training (green) performance on neuropsychological measures **(A)** and PSC computerized cognitive tests **(B)** for the 12 participants that completed the study.

We have found in prior studies that gains in auditory processing speed can serve as a behavioral measure of target engagement, i.e. improvements in basic sensory processing speed best correlate with enhancements in more complex cognition (51, 57). Consistent with this, we found significant improvement in auditory processing speed as measured by the PSC-CC on-line assessment battery, despite the wide range in training dose achieved during the time period available for the study. This finding indicates that participants were actively engaged with the training exercises. Confirming target engagement, we found significant improvements at the group level in two global neuropsychological outcome measures: the Cognition Composite Score and the general Speed of Processing score. These results indicate generalization of training effects to global cognitive operations. Improvements at trend-level were seen in Cognitive Flexibility/Task Switching, while improvements in measures of Working Memory, Attention, Response Inhibition, and Problem Solving were non-significant (**Table 2**, **Figure 3**). Effect sizes were in the large range in the Cognition Composite Score and Speed of Processing Score, and in the small to medium range in Verbal Learning, Working Memory, Cognitive Flexibility/Switching, Problem Solving, and Inhibition (**Table 2**).

**Table 2:** Baseline and post-training performance, and effect sizes with lower and upper confidence intervals in neuropsychological measures and automated computerized cognitive tests.

Neuropsychological Measures	Baseline Mean (SD)	Post Mean (SD)	T-test (p value)	Effect Size *d*	Lower C.I.	Upper C.I.
Cognition Composite Score	36.07 (6.05)	41.52 (6.07)	3.32 (<0.01)	0.90	-0.01	1.74
Attention	38.86 (9.64)	41.10 (8.71)	1.00 (0.34)	0.24	-0.60	1.07
Speed of Processing	39.00 (5.13)	45.49 (8.14)	3.70 (<0.01)	0.95	0.04	1.80
Working Memory	19.29 (7.87)	24.52 (14.31)	1.23 (0.25)	0.45	-0.41	1.28
Verbal Learning	40.30 (8.30)	43.04 (8.59)	1.11 (0.29)	0.32	-0.53	1.15
Cognitive Flexibility/Switching	23.81 (20.41)	32.33 (19.34)	2.05 (0.08)	0.43	-0.43	1.26
Problem Solving	46.06 (13.45)	49.83 (9.04)	1.60 (0.14)	0.33	-0.52	1.16
Response Inhibition	41.30 (11.73)	45.90 (12.94)	1.50 (0.17)	0.37	-0.48	1.20
**Automated Computerized Cognitive Tests (PSC-CC)**						
Auditory Processing Speed	43.29 (4.04)	56.71 (11.34)	3.63 (0.01)	1.58	0.52	2.50
Sustained Visual Attention	24.07 (25.76)	45.67 (12.24)	3.84 (0.00)	1.07	0.09	1.96
Visuospatial Working Memory	35.75 (19.11)	44.84 (8.46)	1.86 (0.10)	0.62	-0.31	1.48
Visual Cognitive Control	41.41 (7.26)	52.43 (6.76)	3.64 (0.01)	1.57	0.51	2.50

Significant improvement was also seen in the PSC-CC battery measures of sustained visual attention and cognitive control, while improvement in visuospatial working memory was non-significant. Effect sizes were in the large range in 3 of the 4 measures and in the medium range for visuospatial working memory (**Table 2**).

### The Effects of Training “Dose”

**Figure 4:**
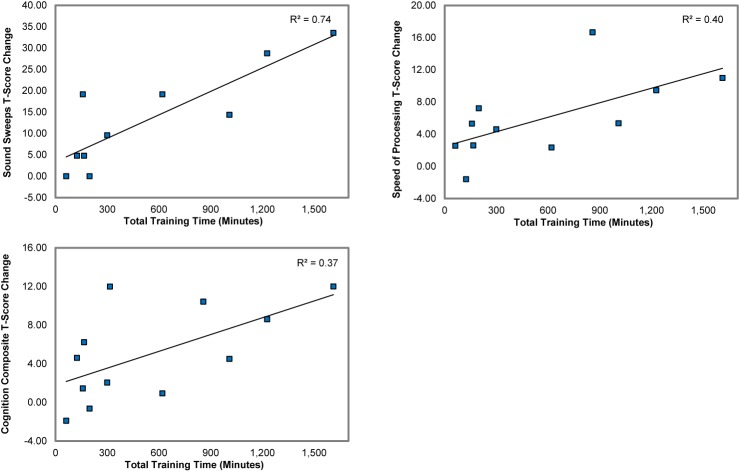
Associations between total training time and the gains in Auditory Processing Speed (Sound Sweeps), Speed of Processing, and the Cognition Composite score.

We found strong and significant associations between total training time and improvement in the measure of target engagement, Auditory Processing Speed, the Speed of Processing measure, and the Cognition Composite Score (.61 < r < .86, .04 < p < .001) (**Figure 4**). Improvement in Auditory Processing Speed was also strongly and significantly associated with gains in the neuropsychological Cognition Composite and Speed of Processing measures (.66 < r < .72, .02 < p < .04). These results indicate that the greater the hours of training, the larger was the training-induced target engagement, and the higher the gains made in global neuropsychological outcome measures.

## Discussion

The results of this pilot study indicate first, that both standardized neurocognitive measures and a computerized cognitive assessment battery can be successfully administered to CD adolescent males incarcerated in a high-security setting for violent crimes. Second, we also found that an intensive course of cognitive training delivered *via* tablets is feasible and tolerable in this high-risk population. Finally, our results demonstrate preliminary efficacy in improving neurocognitive performance in incarcerated CD youth, and show that cognitive gains induced by training are significantly associated with a psychophysical measure of target engagement and with the “dose” of training. Notably, we found that 86% percent of the boys who enrolled in the study were able to complete the assessments, adhere to the cognitive training protocol, and provide post-intervention data. This high level of engagement was undoubtedly due to the unique nature of the MJTC treatment setting, with its close collaboration between mental health and correctional staff, as well as the presence and supervision of a psychologist who is engaged in research activities.

The MJTC youth demonstrated a range of comorbid psychiatric diagnoses in addition to CD. They also exhibited a baseline profile of cognitive impairments across a range of measures, including sustained attention, working memory, and cognitive flexibility/task switching, with scores ranging ~1-3 SD below age-matched norms. Deficits in executive and cognitive control functions involved in problem solving and decision making, such as planning, working memory, inhibition, and cognitive flexibility, have been shown in several studies of CD [for a review, see Matthys et al. (58)]. Our results are also consistent with a study of young individuals with CD, ages 12–21, who had engaged in violent and antisocial behavior (59). The greatest cognitive impairments in Johnson et al., and in our study, were observed in measures of executive function where subjects in both studies performed in the severely impaired range.

Interestingly, we were able to demonstrate that the cognitive impairments in these incarcerated adolescents demonstrated malleability in response to a relatively brief dose of neuroscience-informed cognitive training delivered over a period of eight weeks. The effect sizes suggest that the training may be particularly effective in inducing gains in measures of processing speed, visual attention, visual cognitive control, and global cognition. To a lesser extent, the training shows positive effects in improving working memory, verbal learning, and in other executive functions (i.e. cognitive flexibility, problem solving, and inhibition). Further, the magnitude of the cognitive gains directly associated with the duration of training in which the participants engaged. These findings suggest that gains are not simply a result of practice effects on the neuropsychological tests, of non-specific environmental enrichment due to study participation, or of “placebo effects”. However, these results should be interpreted with caution given the small sample size and lack of a control group.

While these preliminary results are encouraging, there are several limitations to this study. First, we studied a small sample of incarcerated youth in a unique treatment setting, as noted above. Second, the lack of a control condition prevents us from determining whether practice effects were an important factor in our results. However, given the large effect sizes observed for the Cognition Composite, Speed of Processing and 3 of 4 computerized cognitive assessments, as well as the strong associations between cognitive improvement, dose of training, and target engagement, it is unlikely that these results are solely due to the effects of repeated exposure to the neuropsychological and computer-based assessments. Third, we do not know if the profile of cognitive impairment we observed in the MJTC youth is due to poor intrinsic motivation or low effort to complete the assessments—though our observations of the participants suggest that this was not the case. It is also important to note that delivering cognitive training in a maximum security setting presents unique challenges, and that future research may best be served by studying these methods in less restrictive environments. Finally, the hours of training that participants completed was variable. Future research is needed to determine what factors contribute to and may enhance compliance. Despite these limitations, our results indicate that cognitive training is feasible, tolerable, and acceptable to incarcerated youth, and that there is evidence of preliminary efficacy. These data provide support for undertaking well-powered double-blind randomized controlled trials of this intervention.

We do not wish to imply that cognitive training should become, or will become, the sole treatment approach for incarcerated high-risk youth. The gold standard will continue to be evidence-based psychosocial programs that focus on the mental health and rehabilitative needs of these adolescents. However, if a relatively short course of intensive cognitive training on a mobile device can lead to a modest improvement in certain cognitive domains in high risk incarcerated youth—such as global cognition and speed of processing—this might enable some individuals to make better use of educational and vocational rehabilitation programs, thereby supporting better decision-making and more adaptive functioning upon release into the community.

## Data Availability Statement

The datasets generated for this study are available on request to the corresponding author.

## Ethics Statement

This study was approved by the Institutional Review Boards at Medota Juvenille Treatment Center and the University of California San Francisco. Participants provided assent, and a legally-authorized-representative (LAR) provided adult guardian consent.

## Author Contributions

All authors conceived the presented idea, discussed the results and contributed to the final manuscript. AR, BB, and MC carried out the experiment. MF and JM performed computations and verified analytical methods.

## Funding

Funds for this pilot study were provided to Dr. Vinogradov by the Northern California Institute of Research and Education.

## Conflict of Interest

The cognitive training software used in this study was supplied to the corresponding author free of charge by Posit Science. SV serves on Scientific Advisory Boards for the following organizations: Mindstrong, Inc, Alkermes, Inc., and Psyberguide. SV and MF have scientific collaborations with scientists at Post Science, Inc. MN is the co-inventor of the cognitive training software used in the study. She was a paid employee at Posit Science during the study and is now a paid consultant to Posit Science.

The remaining authors declare that the research was conducted in the absence of any commercial or financial relationships that could be construed as a potential conflict of interest.
