# Supplementary material for: Cognitive Training for Very High Risk Incarcerated Adolescent Males
Source: Front Psychiatry. 2020 Apr 15;11:225. doi: 10.3389/fpsyt.2020.00225 (PMC7175987; doi:10.3389/fpsyt.2020.00225)
Supplement: Supplementary file 1 [file Table_1.docx]

**Supplemental Table 1. Targeted Cognitive Training Exercises Completed via iPad.**

| BrainHQ Exercise Name | Description |
| --- | --- |
| AUDITORY EXERCISES | |
| Sound Sweeps | Sound sweep processing: Indicate the direction of two consecutive sound sweeps. Each one can sweep either up or down in pitch |
| Memory Grid | An auditory Memory game: Match pairs of sound cards in a memory game |
| Fine Tuning | A syllable discrimination task: Select the syllable you just heard from a given pair. Syllables in the pair become increasingly similar as training progresses |
| Syllable Stacks | Memorize the syllables you just heard and click on them in the order you heard them. The sequence becomes longer as the task progresses |
| VISUAL EXERCISES | |
| Hawk Eye | Identify the different bird from an array of birds presented peripherally. Presentation duration shortens as the task progresses |
| Target Tracker | A multiple object tracking task: Track moving target objects on the screen among distractors as they shift location |
| Eye for Detail | A spatial working memory task: Identify the two matching objects that rapidly appear on the screen in various spatial locations |
| Double Decision | A speeded UFOV dual task: Correctly discriminate the vehicle in the center of the screen AND identify the location of the road sign appearing briefly in the periphery. |
| Visual Sweeps | Indicate the direction of two consecutive moving visual sweeps. Each one can sweep either inward or outward |
